# First satellite tracks of South Atlantic sea turtle ‘lost years’: seasonal variation in trans-equatorial movement

**DOI:** 10.1098/rspb.2017.1730

**Published:** 2017-12-06

**Authors:** Katherine L. Mansfield, Milagros L. Mendilaharsu, Nathan F. Putman, Maria A. G. dei Marcovaldi, Alexander E. Sacco, Gustave Lopez, Thais Pires, Yonat Swimmer

**Affiliations:** 1 Marine Turtle Research Group, University of Central Florida, Orlando, FL 32816, USA; 2 Projeto TAMAR/Fundação Pró-TAMAR, Salvador, BA, Brazil; 3 LGL Ecological Research Associates, Inc., Bryan, TX 77801, USA; 4 Atlantic Oceanographic and Meteorological Laboratory, National Oceanic and Atmospheric Administration, Miami, FL 33149, USA; 5 NOAA Fisheries, Pacific Islands Fisheries Science Center, Honolulu, HI 96818, USA

**Keywords:** *Caretta caretta*, marine turtle oceanic stage, dispersal, population connectivity, ocean currents, South Atlantic Ocean

## Abstract

In the South Atlantic Ocean, few data exist regarding the dispersal of young oceanic sea turtles. We characterized the movements of laboratory-reared yearling loggerhead turtles from Brazilian rookeries using novel telemetry techniques, testing for differences in dispersal during different periods of the sea turtle hatching season that correspond to seasonal changes in ocean currents. Oceanographic drifters deployed alongside satellite-tagged turtles allowed us to explore the mechanisms of dispersal (passive drift or active swimming). Early in the hatching season turtles transited south with strong southward currents. Late in the hatching season, when currents flowed in the opposite direction, turtles uniformly moved northwards across the Equator. However, the movement of individuals differed from what was predicted by surface currents alone. Swimming velocity inferred from track data and an ocean circulation model strongly suggest that turtles' swimming plays a role in maintaining their position within frontal zones seaward of the continental shelf. The long nesting season of adults and behaviour of post-hatchlings exposes young turtles to seasonally varying ocean conditions that lead some individuals further into the South Atlantic and others into the Northern Hemisphere. Such migratory route diversity may ultimately buffer the population against environmental changes or anthropologic threats, fostering population resiliency.

## 1. Background

Understanding dispersal and behaviour of a species throughout its life cycle is critical for species conservation [1–3]. Early life-history data are particularly challenging to collect among highly migratory and long-lived marine animals, owing to their small size and the inaccessibility of the open ocean [2,4]. For sea turtles, the in-water dispersal of hatchlings from their natal beaches, and subsequent movements and behaviour during their first years at sea (aptly named the sea turtle ‘lost years’) remains largely a mystery [4,5]. Early sea turtle ecology and demography are among the most important gaps in sea turtle population assessments [2,6].

The prohibitive cost and logistics associated with offshore sampling historically limited early sea turtle life-history data to opportunistic sightings [7–9], acquiring genetic and size data from turtles accessible from islands such as the Azores or Cape Verde Islands [10–12], laboratory-based experiments studying the sensory ecology of hatchlings [13,14], or simulations of dispersal in ocean circulation models [15,16]. The challenges associated with sampling broadly dispersed, cryptic species in the open ocean resulted in a ‘patchwork’ of data and observations that formed the foundation of accepted life-history and population models persisting for decades. This ‘patchwork’ of inferred data suggests that most species remain at the sea surface within offshore, oceanic (greater than 200 m depth) waters [7–9,17,18], associate with *Sargassum* or other flotsam [8,9,18,19], and passively drift and entrain within prevailing ocean currents such as those associated with the North Atlantic Subtropical Gyre (NASG) (e.g. [8,9]). Laboratory work demonstrated that young loggerhead sea turtles (*Caretta caretta*) in the North Atlantic have an innate magnetic sense [13,14], orienting using the Earth's magnetic field as a map to remain within the boundaries of the NASG [20]; however, empirical in-water observations or surveys throughout the ocean basin were missing.

Recently, studies using small, solar-powered bird satellite tags provided insight on the *in situ* movements and behaviour of sea turtles during their ‘lost years’ [5,21]. This work documents that not all young, oceanic stage turtles behave as historically hypothesized [5]. While the tracked turtles travelled offshore of the continental shelf and probably remained at or near the sea surface, many turtles dropped out of the currents associated with the Gulf Stream System into the interior of the gyre and the Sargasso Sea, exhibiting strong directional movement [5]. Further, surface- and *Sargassum-*dwelling young turtles probably benefit from solar radiation and absorption while basking at the sea surface or perched on top of brown mats of *Sargassum* [5]*.* This behaviour and a newly hypothesized thermal niche have considerable implications for the survival, fitness and physiology of small, ectothermic surface-dwelling animals dispersing at sea. These findings prompt new questions: is this behaviour shared among sea turtles from different rookeries within different ocean basins where floating habitats such as *Sargassum* may not be available, or where sea surface temperatures may vary from those in the North Atlantic? Gulf of Mexico field experiments suggest that young wild-caught oceanic turtles are active swimmers and that there are differences among species in this behaviour [22]. These results have broad implications on survival estimates and genetic connectivity for species originating in different regions. Until recently, few empirical studies occurred outside of the western North Atlantic (but see [23–25]), yet data collected within this region were widely applied to all species in all ocean basins (e.g. as reviewed in [17,18]).

In the South Atlantic Ocean, very few empirical data exist regarding the early dispersal and in-water behaviour of oceanic juveniles beyond the hatchling and post-hatchling stage. This ocean basin contains a gyre system similar to that in the North Atlantic, where the Gulf Stream runs offshore of the North Atlantic's primary sea turtle nesting beaches along the Atlantic US coast. The South Equatorial Current (SEC) forms the northern part of the South Atlantic Ocean Subtropical Gyre (SASG), carrying subtropical water from the Benguela Current region towards the Brazil shelf region around 14° S, where, unlike the Gulf Stream in the western North Atlantic, it bifurcates into two western boundary currents (WBCs): the Brazil and North Brazil Currents (BC and NBC) [26]. In the South Atlantic, loggerhead nesting is concentrated along the coast of Brazil, with the majority of nests (approx. 5000 annually) occurring within Bahia, Brazil [27]. Long- and near-shore currents in the western South Atlantic occur offshore of these primary loggerhead nesting beaches; yet, these currents differ from the Gulf Stream in the North Atlantic in that seasonal changes in these currents occur proximal to Brazilian sea turtle nesting beaches, particularly off the Bahia coast to the north. These seasonal changes in ocean circulation could have far-reaching implications for hatchling dispersal and rookery productivity associated with Brazil's loggerhead populations.

**Figure 1.**
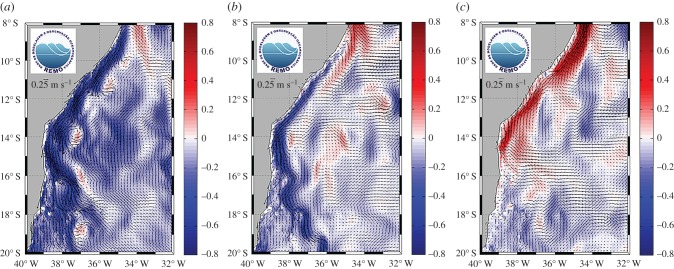
(*a*–*c*) Prevailing currents offshore of Bahia, Brazil, sea turtle rookery (*a*) early-hatching season (7 December), (*b*) mid-hatching season (4–8 March) and (*c*) late-hatching season (1 May). Mean horizontal surface currents (represented by vectors) derived from Global HYCOM [30] for (a) 7 December (early-hatching season), (*b*) 4–8 March (mid-hatching season) and (*c*) 1 May (late-hatching season). The colours represent the intensity (m s^−1^) of the meridional component (or *y*-coordinate) of the velocity, where positive values (red) represent a current towards the Equator and negative values (blue) represent a current towards the south. Source: REMO Ocean Data Assimilation System (RODAS) [31].

In Brazil, the loggerhead nesting season occurs from September to March, peaking in November and December [28,29], with hatchlings emerging from nests November through May. It is during this period of hatchling emergence that the intensity and direction of ocean currents off the Bahia coastline change from southward to northward as the hatching season progresses (figure 1*a*–*c*), with the additional formation of mesoscale eddy features spinning off of the currents in the mid-hatching season [32].

The continental shelf off the central Brazil coast, extending from 10°S to 16°S, is a unique region because it not only hosts the narrowest shelf area along the eastern South American coast [33], but it is also located where the bifurcation of the South Equatorial Current (biSEC) takes place, creating the two WBCs: the poleward BC and the equatorward NBC [26,33]. Annually, the biSEC occurs on average between approximately 10° S to 14° S [34]. Recent ocean circulation studies note that there is significant seasonal variation in the latitudes of the bifurcation, reaching its most northerly position at 12° S in December and its most southerly position at 17° S in July [35]. Numerical ocean circulation models predict that hatchlings emerging from Brazilian nesting beaches seasonally disperse to the north along the coast within the North Brazil Current and to the south within the Brazil Current [4,36,37].

Satellite tracking studies of nesting female loggerheads from Bahia, Brazil, reveal that post-nesting, some turtles migrate north, while others go south towards neritic foraging areas off the N-NE and S-SE coast of Brazil [38]. Genetic studies, mark–recapture data and by-catch data of loggerhead turtles (adults and subadults) within neritic waters off south Brazil and the Rio de la Plata estuary indicate that these turtles originated from Brazilian rookeries including Bahia, Espírito Santo and Rio de Janeiro [39–41]. Genetic analyses of juvenile loggerheads incidentally captured in pelagic longlines in the Rio Grande Seamount show that 59.5% of these juveniles originated from Brazilian rookeries, and 40.5% belonged to other populations (North Atlantic, Mediterranean and Indo-Pacific) [42–44]. Primary foraging grounds for larger juveniles and subadults are located along the continental shelf and slope within the Brazil, Uruguay and Argentina Exclusive Economic Zones and adjacent international waters [42,45]. Juvenile loggerheads also occur in neritic and oceanic waters off the NE coast of Brazil, though less frequently than in the south [42,43]. Small juveniles (less than 39 cm) are observed within Brazilian waters; however, initial dispersion patterns and early life history of loggerheads in the South Atlantic largely remains a mystery.

Here, we use an empirical and theoretical approach to (i) characterize the satellite-tracked movements and dispersal of oceanic stage loggerhead turtles in the southwestern Atlantic Ocean, (ii) test for differences in dispersal patterns during different periods (early, middle and late) of the sea turtle hatching season, (iii) experimentally test and model whether the turtles passively drift with local ocean currents or actively disperse (per [22]), and (iv) compare the behaviour and dispersal of young oceanic stage loggerheads in the Northwest Atlantic (e.g. [5]) to those in the Southwest Atlantic.

## 2. Material and methods

### (a) Tagging and tracking of sea turtles and drifters

We deployed 19 solar-powered (9.5 g Microwave Telemetry, Inc.) satellite tags on laboratory-reared loggerhead sea turtles hatched from Bahia nesting beaches in November 2011 (*n* = 14) and April 2012 (*n* = 5). Turtles were reared to sizes greater than 10 cm straight carapace length (SCL) per Mansfield *et al*. [21] to ensure the tags were less than 3–5% of the turtles' weight. Tags were attached to the turtles’ carapaces using a flexible acrylic-silicone-neoprene attachment method described by Mansfield *et al*. [5,21]. At release, turtle age averaged 230.1 d (±110.5 d s.d.; range = 123–401 d), and SCL averaged 18.0 cm (±4.6 cm s.d.; range = 10.8–26.8 cm; table 1).

**Table 1.** Metadata for the satellite-tracked turtles including individual tag ID; age of turtle (days), standardized straight carapace length (SCL) measured from notch to tip (n-t) measured in centimetres; date and location (latitude/longitude) of release; and number of days tracked post-release.

turtle ID	age (days)	SCL (n-t)	release date	latitude	longitude	track days	Argos positions (no.)
*121364a*^b^	221^a^	10.80	8 Nov 2012	−12.645	−37.927	14	16
*121367*	224^a^	16.80	11 Nov 2012	−12.637	−37.942	65	196
*121369*	224^a^	14.30	11 Nov 2012	−12.650	−37.940	5	19
*21364*	338^a^	14.20	4 Mar 2013	−12.658	−37.912	120	271
*121366*	338^a^	19.30	4 Mar 2013	−12.658	−37.914	43	86
*121371*	123	15.20	4 Mar 2013	−12.658	−37.914	68	179
*102119*	127	13.70	8 Mar 2013	−12.694	−37.934	49	110
*102145*	127	13.70	8 Mar 2013	−12.692	−37.279	87	334
*121365*	181	17.30	1 May 2013	−12.645	−37.882	45	178
*121363*	181	18.20	1 May 2013	−12.645	−37.882	56	184
*121370*	181	16.70	1 May 2013	−12.645	−37.882	49	134
*102146*	181	17.20	1 May 2013	−12.644	−37.881	62	226
*102150*	181	17.40	1 May 2013	−12.644	−37.881	68	292
*107875*	181	17.00	1 May 2013	−12.644	−37.881	45	145
*121368*	181	17.50	1 May 2013	−12.645	−37.882	51	174
*102116*	401	25.60	7 Dec 2013	−12.618	−37.903	35	38
*102117*	401	26.30	7 Dec 2013	−12.618	−37.903	31	51
*102123*	377	26.80	7 Dec 2013	−12.618	−37.903	26	57
*102126*	401	24.10	7 Dec 2013	−12.618	−37.903	29	51

^a^Hatch date estimated.

^b^These tags were attached to the same turtle. This turtle, stranded during the first release, was rehabilitated and raised to a larger size before being released in the mid-hatching season.

Six releases, with 1–4 turtles per release, occurred at different times throughout the hatching season, following predicted changes in current patterns off the Bahia coast. The prevailing current flows south in the early- to mid-hatching season and north in the late-hatching season; turtles were released in November/December (*n* = 7; 2012 and 2013), March (*n* = 5; 2013), and May (*n* = 7; 2013) to span the entirety of the hatchling dispersal period. Turtles were released close to the continental shelf slope, 10 km from the coast within the prevailing currents. With each turtle release, we deployed passive oceanographic drifters to serve as controls in testing for active versus passive turtle behaviour [22]. Following the methods of Putman & Mansfield [22], two types of drifters were deployed at the same location and times as the turtles were released: surface ‘Eddie’ drifters with drogues extending to 1 m depth (*n* = 4) and very-near surface ‘Kathleen’ or ‘Bruno’ bucket drifters that remain in the upper 0.37 m of the water column (*n* = 10).

Location data from turtles were imported into the Geographic Information System (GIS) and seaturtle.org's Satellite Tracking and Analysis Tool [46] to filter out location error. Location data from the satellite tags were derived from Argos location data and were archived and filtered using standard methods [5,47]. Positional data were further extracted from tracks of turtles at approximately 48 h intervals (steps) using only the best quality Argos location data (classified as ‘0’, ‘1’, ‘2’ or ‘3’, for which location errors are typically less than 5 km [48–50]). This sub-sampling of data allowed for standardization of track data and the number of steps used in subsequent analysis. We obtained 323 steps from the 19 loggerhead tracks. Eddie and Kathleen drifter track data were pooled for analysis and positional data were sub-sampled at approximately 48 h intervals [22], resulting in 213 steps from 14 drifters.

To provide additional oceanographic context to the tracking data, we performed simulations using the surface layer of the Global Hybrid Coordinate Ocean Model (HYCOM) [30]. This HYCOM output comprised daily snapshots at 0.08° spatial resolution, obtained from http://hycom.org/. A rectangle (0.08° × 0.08°, approx. 8–9 km) centred at the latitude and longitude of each deployment location served as a release site for 1000 virtual particles. The duration of particle advection was determined by the duration of the longest turtle track from a particular release site. Particles were advected at 30 min intervals through the HYCOM output using the Runge–Kutta fourth-order method applied in ICHTHYOP v. 2 particle-tracking software [51].

### (b) Assessing the role of ocean currents, winds and swimming behaviour on turtle movement

To assess the role of abiotic factors and swimming behaviour on turtle movement, our approach followed methods established by Putman & Mansfield [22]. A 0.08° × 0.08° rectangle (approx. 8–9 km), centred at the latitude and longitude of each approximately 48 h location or time step along the tracks, served as the release site of 200 virtual particles within the surface layer of Global HYCOM output (daily snapshots at 0.08° spatial resolution). This area was chosen to account for any error in location data [16]. The duration of particle advection was determined by the duration between successive points along the track; particles were advected at approximately 15 min intervals through the HYCOM output using the Runge–Kutta fourth-order method applied in ICHTHYOP v. 2 particle-tracking software [51]. The particle closest to the next point along the track was used to calculate the apparent ocean current velocity, derived from the straight-line distance between the starting location of the particle and its end location [22]. The particle vector was subtracted from the track vector (also derived from the straight-line distance between successive locations) to compute the apparent swimming velocity.

Any difference between the track vector and the particle vector is attributed to swimming behaviour; however, divergence is also expected due to incomplete resolution of all factors that might influence an organism's movement at the ocean surface [52,53]. To test whether wind effects not represented in Global HYCOM could be responsible for apparent swimming behaviour, we extracted satellite-derived daily averaged wind velocity along each track. We determined whether ‘swimming’ velocities of turtles were correlated with data from the NOAA Blended Sea Winds (https://www.ncdc.noaa.gov/thredds/OceanWinds.html) using Spearman correlations (for speed) and circular–circular correlations (for direction).

We performed each analysis for the drifters deployed to determine the sensitivity of our numerical methods for inferring behaviour [22,52,53]. We hypothesized that if divergence along the tracks of turtles was primarily the result of model error, Mann–Whitney *U* tests would find no difference between the ‘swimming speeds’ of turtles and drifters [22].

## 3. Results

### (a) Seasonal variation in turtle and drifter movements

**Figure 2.**
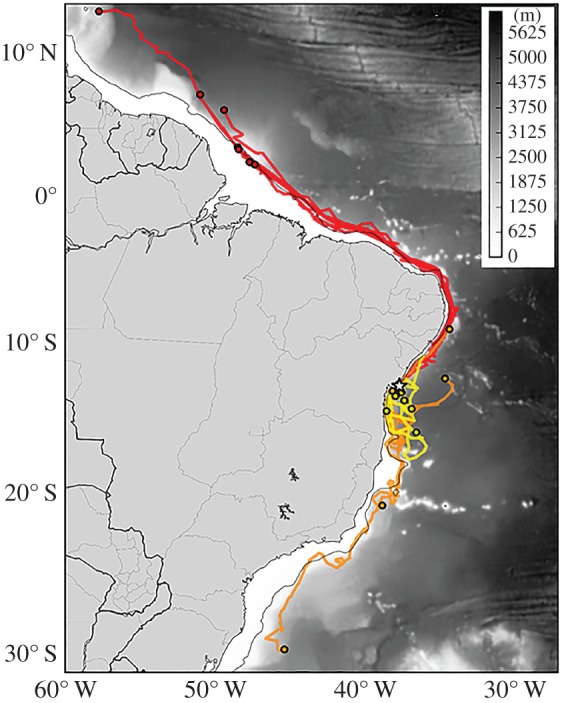
Satellite tracks of yearling loggerhead sea turtles released in the early (yellow), middle (orange) and late (red) hatching season. Star indicates turtle release sites; coloured circles indicate the final position of each track. Grey shading indicates bathymetry, with the thin black line delineating the continental shelf. Laboratory-reared turtles from the same nests/clutches were released early in the hatching season (*n* = 3, November 2012 and *n* = 4, December 2013), in the middle of the hatching season (*n* = 5, March 2013), and late-hatching season (*n* = 7, May 2013). See electronic supplementary material for further oceanographic context.

Turtles were tracked between 5 and 120 d post-release (mean: 49.9 ± 26.2 d s.d.; table 1), ranging between 40 and 4350 km from their release site, travelling as far north as the island of Barbados in the Caribbean, or south off the coast of Rio Grande do Sul in Brazil. Most turtles (*n* = 17) remained within the Exclusive Economic Zone waters of Brazil for the duration of their tag transmissions, while only two travelled outside of Brazil's jurisdictional waters into waters off of French Guyana and Barbados (figure 2; electronic supplementary material, figure S1a–c).

Of the seven turtles released early in the hatching season (November and December), five displayed net southward movement during the period they were tracked (71%), one moved northeast (14%) and one moved west, stranding onshore (14%). Of the six drifters deployed alongside these turtles, four initially drifted south (67%) and two drifted northwest (33%). All drifters travelling northwest washed ashore within approximately 1 day and three south-moving drifters washed ashore within approximately one month. Virtual particles released in the surface layer of Global HYCOM at the same locations and dates as the turtle-drifter deployments travelled south of the release site, mostly remaining on or near the continental shelf (figure 2; electronic supplementary material, figure S1a).

All five turtles released in the middle of the hatching season (March) initially moved southwards; three eventually moved north of the release latitude by mid-May (60%), with one moving offshore and the others remaining along the margin of the continental shelf. The two turtles continuing southwards generally remained just seaward of the continental shelf, both reaching latitude 20° S by June where transmission for one turtle was lost (40%). The other turtle was tracked for an additional month and continued southwards to 30° S. All four drifters simultaneously deployed initially travelled south and beached in less than one month (100%). Similarly, virtual particles tracked during the same period and for the same duration within Global HYCOM were primarily advected southwards and shorewards, with some eventually drifting north over the continental shelf (figure 2; electronic supplementary material, figure S1b).

All seven turtles released at the end of the hatching season (May) quickly moved north along the continental shelf, crossing the Equator between 9 June and 25 June (100%). The longest tracked turtle from this group (final transmission on 8 July) travelled to 13°N, just east of Barbados. All four drifters deployed with these turtles initially travelled north, but beached in less than 2 days (100%). Virtual particles released during this period in Global HYCOM also rapidly travelled northwards and shorewards (electronic supplementary material, figure S1c).

### (b) The role of ocean currents, winds and behaviour

**Figure 3.**
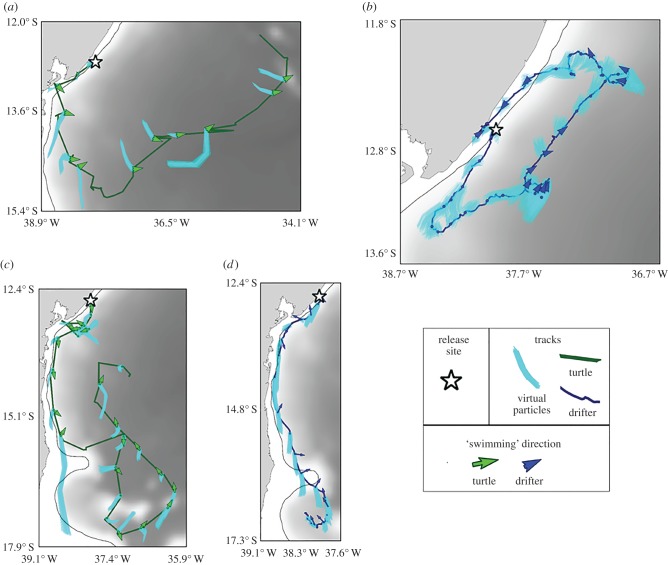
(*a*–*d*) Examples of individual tracks of turtles and drifters, deployed near the continental shelf of Brazil relative to modelled ocean currents. Arrows indicate the calculated swimming velocity along each approximately 48 h track segment. Speeds less than 0.15 m s^−1^ are shown as small circles. Light blue lines along the track indicate the paths of 200 virtual particles released at the corresponding location and time within Global HYCOM output, tracked for approximately 48 h. The white star indicates the release site. Bathymetric scale as in figure 2. Panels show sample movements of individual turtles (*a* = tagID 121366, *c* = tagID 121367) and drifters (*b* = drifterID 320278, *d* = drifterID 950263). Though top and lower panels show some similarities in net movement between turtles and drifters, comparison of tracks relative to particle movements simulated in modelled surface currents indicate that drifter and particle trajectories were in much better agreement than turtle and particle trajectories. These results imply that fine-scale movements of turtles along the coast of Brazil are not entirely driven by ocean circulation processes. The apparent swimming behaviour of turtles can result in substantial differences in net movements between turtles and currents over time (electronic supplementary material, figure 1).

Estimates of ocean currents and winds along turtle and drifter tracks suggest that turtle movements are unlikely to be exclusively driven by ocean currents and winds. Regardless of the release group, ocean currents experienced by turtles and drifters tended to be westwards (median = 270°, Rayleigh *r* = 0.338, *p* = 0, *n* = 535 for turtle and drifter positions, combined) and winds were strongly westwards (median = 265°, Rayleigh *r* = 0.771, *p* = 0, *n* = 518 for turtle and drifter positions, combined). This resulted in drifters consistently washing ashore (93%), but not turtles (only 5%; figure 3*a*,*c*,*d*).

Calculated swimming velocity showed that turtles tended to orient to the northeast (median heading = 52°, Rayleigh *r* = 0.244, *p* = 4.72 × 10^−9^; *n* = 322) at a median speed of 0.262 m s^−1^. Drifter travel direction was also biased eastwards (meaning the actual movement of drifters was less westwards than predicted by HYCOM) (median heading = 89°, Rayleigh *r* = 0.189, *p*= 0.0005; *n* = 213). However, these speeds were much lower than those calculated for turtles (median speed = 0.097 m s^−1^; Mann–Whitney *U* = 13 442, *p* < 0.001; *n* = 322 turtle steps, *n* = 196 drifter steps). No relationship was observed between the calculated swimming speed of turtles and the wind speed (Spearman *r* = −0.02, *p* = 0.975, *n* = 322) or between turtles' swimming direction and wind direction (circular–circular correlation *r* = −0.01, *p* > 0.05, *n* = 322). By contrast, we detected a relationship between the calculated swimming speed of drifters and wind speed (Spearman *r* = 0.185, *p* = 0.009, *n* = 196), though the travel direction of drifters and the direction of wind were unrelated (circular–circular correlation *r* = 0.03, *p* > 0.05, *n* = 196). Thus, the divergence between turtle tracks and modelled ocean currents is not likely attributable to winds or model error, but is most probably due to oriented swimming (e.g. figure 3*a*,*c*).

## 4. Discussion and conclusion

This study represents the first satellite tracks of oceanic stage, or ‘lost years’, sea turtles in the South Atlantic. Changes in turtle movement patterns occurred in conjunction with the seasonal shift in the bifurcation of the SEC into the BC and NBC to the north. In the early-hatching season, this bifurcation occurs to the north of the release sites off of Praia do Forte (12° S), resulting in greater southward flow and transport, while in the late-hatching season, the bifurcation shifts to the south, resulting in northward transport. Track and drifter data suggest that the net movement of turtles is an interaction between turtle behaviour (e.g. orientation and swimming) and ocean circulation processes. Turtles tended to move north or south in response to changes in surface currents; yet, apparent eastward swimming kept turtles from being advected back on to the continental shelf (as occurred with drifters and modelled particles). Such oriented swimming is likely to be adaptive in facilitating their oceanic migration [54,55]. However, in Brazil this behaviour also exposes the young turtles to dynamic and seasonally varying ocean conditions. Such seasonal variation in oceanic dispersal has been predicted for sea turtle populations in other regions, including leatherback (*Dermochelys coriacea*) rookeries in the eastern South Atlantic Ocean (e.g. [56]). For Brazil's loggerheads in the western South Atlantic, tracking data indicate that spatio-temporal variation in early dispersal may result in a diversity of movement types that lead some individuals into the Northern Hemisphere, and others further into the South Atlantic.

Turtles in this study were laboratory-reared to a size appropriate for satellite tagging (per [21]), so it is possible that these turtles may behave differently than they would had they entered the ocean directly after hatching, or had been captured in the wild. However, young loggerheads are documented to have an innate magnetic compass and map sense (per [13,14]) and would be expected to orient to remain within waters hospitable to their growth and survival. Regardless, the highly divergent dispersal trajectories observed imply that older juveniles would need to possess a robust navigational system to return to their natal coast upon reaching maturity, as it does not appear that ocean currents would readily return turtles to the coast of Brazil [36].

We observed similar track durations for those turtles tracked in the Mansfield *et al*. [5] North Atlantic study. Turtle trajectories in the South Atlantic were mostly parallel to the coast, travelling along the outer edge of the continental shelf (for turtles travelling north or south), while those entrained in the eddy field moved further into oceanic waters. In the North Atlantic, all turtles initially dispersed within the Gulf Stream, the WBC of the NASG [5]. While some North Atlantic turtles departed the Gulf Stream towards the oceanic waters of the Sargasso Sea after their initial dispersal, all turtles remained entrained within the confines of the NASG and initial net dispersal of all tracked North Atlantic turtles was to the north/northeast. In this study, only turtles released early- and in the mid-hatching season remained associated with or within the SASG. None of the late-season releases remained within the gyre system, exiting the SASG in the NBC. Further, turtle dispersal trajectories changed based on seasonal shifts in local current patterns, a phenomenon that does not occur in the North Atlantic's Gulf Stream or adjacent to the North Atlantic's primary loggerhead nesting beaches. Young turtles in the western South Atlantic appear to be influenced by seasonal changes in these currents, travelling south or remaining in the eddy field early in the hatching season, and travelling north along the coastline in the late season on trajectories bringing late-season turtles into the Northern Hemisphere. Thus, seasonal trans-equatorial transport of oceanic stage turtles is possible and probably results in contributions of Brazilian turtles to mixed-stock foraging grounds in the North Atlantic [12].

Turtles tracked crossing the Equator were rapidly travelling northwards with the North Brazil and Guiana Currents when transmission was lost. These trajectories probably would bring turtles through the Caribbean Sea, Gulf of Mexico, and possibly into the NASG. Our findings support genetic data showing that a relatively large percentage of Brazil's turtles occur in the northeastern Atlantic [12] and, more generally, the broad connectivity across ocean basins that is predicted in ocean modelling studies (e.g. [4,57]). Based on seasonal variation in dispersal trajectories observed (figure 2) and the proportion of hatchlings that emerge each month [28,29], approximately 75% of Brazilian turtles would be expected to remain in the Southern Hemisphere, whereas 25% of the population would travel into the Northern Hemisphere.

It remains unanswered whether there are differences in sex ratios (differences in incubation temperatures early versus late season) or survival and growth for the dispersal trajectories observed (those remaining in northern habitats with year-round favourable temperatures might have increased fitness compared with those moving further south). These factors have important implications for understanding mixed-stock foraging aggregations and meta-population dynamics in sea turtle populations.

Female loggerheads nesting in Bahia lay an average of four nests per reproductive season, with individuals laying as many as eight observed nests per season [58]. So, it is probable that an individual female could lay clutches that would result in her offspring/genetics dispersing to *both* southern and northern regions, thereby not ‘putting all of her eggs in one hemispheric basket’. Dynamic oceanic conditions offshore of the Bahia rookery may select for plasticity in behavioural responses among young oceanic turtles—depending on the abiotic conditions encountered or experienced, turtles may behave differently.

Dynamic conditions may not allow turtles to channelize on a single ‘preferred’ oceanic region for their nursery habitat; however, this may buffer the population against environmental changes or even anthropogenic threats in different regions, fostering population resiliency versus population abundance [59].

Many questions remain regarding the early life history of sea turtles. The need for improved tracking technologies is evident. No methods yet exist to track these animals over the entirety of their long (greater than 1–2 years) oceanic stage. Miniaturized ‘nano-tags’ have been used to track hatchling sea turtles as they depart the nesting beach, but these only provided data for the first approximately 15 km (approx. 8 h) of their transoceanic migration [60]. The tags in this study transmit data over longer periods and larger areas, but are too large to affix to hatchlings [21]. Additionally, these tags have duty cycles that limit transmissions to less than 12 h per 48 h recharging cycles. Smaller, more accurate (e.g. GPS capable) satellite tags are needed with 24 h transmissions to allow detailed insight into turtles' activity including the proportion of time turtles are actively swimming or orienting versus at rest, and whether there are diel patterns of behaviour. Other small marine swimmers are known to diverge from the direction of local currents or water flow [61]; incorporating compass headings with swim speeds would help refine our understanding of the degree to which (and when) young, oceanic turtles are moving with, or independently of, local currents [62]. As more laboratory-reared and wild-caught oceanic stage sea turtles are tracked from different rookeries in different ocean basins, we will probably find that long-held hypotheses regarding the ‘lost years’ dispersal and behaviour (e.g. that turtles passively drift within ocean gyre currents [8,9]) cannot be applied to all turtles everywhere—one hypothesis does not fit all.

## Supplementary Material

Supplemental Figure 1

## Data accessibility

Track data associated with this study are archived online on seaturtle.org's Satellite Tracking and Analysis Tool [46] and Dryad Digital Repository (http://dx.doi.org/10.5061/dryad.7fh30) [63].
