# Supplementary material for: First satellite tracks of South Atlantic sea turtle ‘lost years’: seasonal variation in trans-equatorial movement
Source: Proc Biol Sci. 2017 Dec 6;284(1868):20171730. doi: 10.1098/rspb.2017.1730 (PMC5740273; doi:10.1098/rspb.2017.1730)

**First satellite tracks of South Atlantic sea turtle ‘lost years’: seasonal variation in trans-equatorial movement**

Katherine L. Mansfield^1^*

Milagros L. Mendilaharsu ^2^

Nathan F. Putman^3,4^,

Maria A. G. dei Marcovaldi^2^

Alexander E. Sacco^1^,

Gustave Lopez^2^

Thais Pires^2^

Yonat Swimmer^5^

^1^Marine Turtle Research Group, University of Central Florida, Orlando, Florida 32816 USA.

^2^Projeto TAMAR-ICMBio and Fundação Pró-TAMAR, Salvador, BA, Brazil.

^3^ LGL Ecological Research Associates, Inc., Bryan, Texas 77801, USA

^4^Atlantic Oceanographic and Meteorological Laboratory, National Oceanic and Atmospheric Administration, Miami, Florida 33149 USA

^5^NOAA Fisheries, Pacific Islands Fisheries Science Center, Honolulu, Hawaii USA 96818

*Corresponding Author: [kate.mansfield@ucf.edu](mailto:kate.mansfield@ucf.edu)

**SUPPLEMENTAL DATA**

**Supplemental Figure 1.** Satellite tracks of oceanic stage laboratory-reared loggerhead sea turtles released in the (A) early (yellow), (B) middle (orange), and (C) late (red) hatching season off the coast of Bahia, Brazil in the western South Atlantic. Stars indicate the release site of turtles; colored circles indicates the final position of each track. Turtle tracks are overlaid on tracks of simultaneously deployed drifters (dark blue lines) and virtual particles released in the surface layer of Global HYCOM at the corresponding time and location of turtle/drifter deployments. Gray shading indicates bathymetry with the thin black line delineating the continental shelf. Lab-reared turtles from the same nests/clutches were released (B) early in the hatching season (n=3, November 2012 and n=4, December 2013), (C) in the middle of the hatching season (n=5, March 2013), and (D) late in the hatching season (n=7, May 2013). Comparison of turtle tracks to those of drifters and particles indicates that the movement of young sea turtles differs substantially than what would be predicted by ocean currents alone.


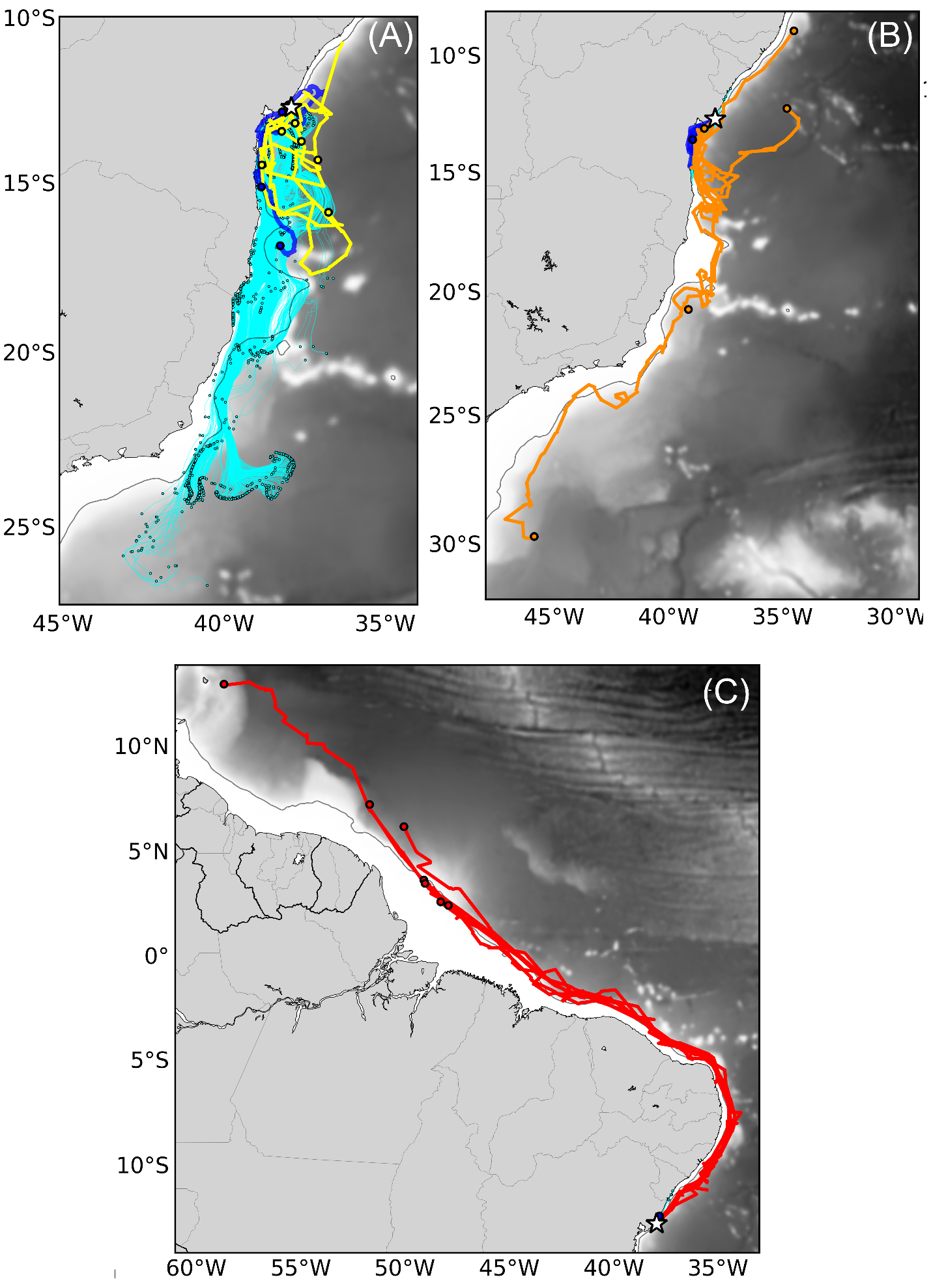

Supplement: Supplemental Figure 1 [file rspb20171730supp1.docx]
